# Volume overload is a major characteristic in primary aldosteronism: a 3-year follow-up study

**DOI:** 10.1097/HJH.0000000000003696

**Published:** 2024-02-21

**Authors:** Eeva Kokko, Manoj Kumar Choudhary, Aapo Mutanen, Milja Honkonen, Antti Tikkakoski, Jenni K. Koskela, Mari Hämäläinen, Eeva Moilanen, Marianna Viukari, Niina Matikainen, Pasi I. Nevalainen, Ilkka Pörsti

**Affiliations:** a Faculty of Medicine and Health Technology, Tampere University; b Department of Clinical Physiology and Nuclear Medicine; c Department of Internal Medicine, Tampere University Hospital; d Immunopharmacology Research Group, Tampere University and Tampere University Hospital, Tampere; e Endocrinology, Helsinki University Hospital and Research Programs Unit, Clinical and Molecular Medicine, University of Helsinki, Helsinki, Finland

**Keywords:** adrenalectomy, extracellular water volume, haemodynamics, primary aldosteronism, spironolactone

## Abstract

**Objectives:**

We examined haemodynamics, focusing on volume balance and forward and backward wave amplitudes, before and after 2.8 years of targeted treatment of primary aldosteronism. Patients with essential hypertension and normotensive individuals were examined for comparison (*n* = 40 in each group).

**Methods:**

Recordings were performed using radial artery pulse wave analysis and whole-body impedance cardiography. Unilateral aldosteronism was treated with adrenalectomy (*n* = 20), bilateral aldosteronism with spironolactone-based medication (*n* = 20), and essential hypertension with standard antihypertensive agents.

**Results:**

Aortic SBP and DBP, forward and backward wave amplitudes, and systemic vascular resistance were equally elevated in primary aldosteronism and essential hypertension. All these haemodynamic variables were similarly reduced by the treatments. Primary aldosteronism presented with 1 litre (∼10%) extracellular water excess (*P* < 0.001) versus the other groups, and this excess was normalized by treatment. Initial pulse wave velocity (PWV) was similarly increased in primary aldosteronism and essential hypertension, but final values remained higher in primary aldosteronism (*P* < 0.001). In regression analyses, significant explanatory factors for treatment-induced forward wave amplitude reduction were decreased systemic vascular resistance (*β* = 0.380) and reduced extracellular water volume (*β* = 0.183). Explanatory factors for backward wave amplitude reduction were changes in forward wave amplitude (*β* = 0.599), heart rate (*β* = −0.427), and PWV (*β* = 0.252).

**Conclusion:**

Compared with essential hypertension, the principal haemodynamic difference in primary aldosteronism was higher volume load. Volume excess elevated forward wave amplitude, which was subsequently reduced by targeted treatment of primary aldosteronism, along with normalization of volume load. We propose that incorporating extracellular water evaluation alongside routine diagnostics could enhance the identification and diagnosis of primary aldosteronism.

## Graphical abstract

**Figure:**
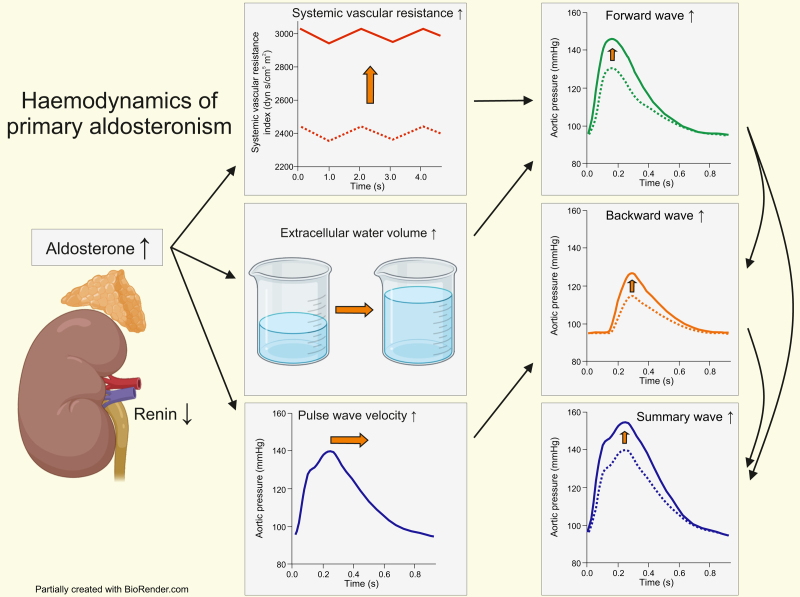

## INTRODUCTION

Primary aldosteronism is the most common form of secondary hypertension [1]. Targeted treatment reduces cardiovascular complications in primary aldosteronism [2], but often the condition remains unrecognized even among high-risk patients [3,4]. Primary aldosteronism screening is largely based on the determination of aldosterone-to-renin ratio (ARR), but improved diagnostic approaches are needed [1,4].

Aldosterone plays a key role in the control of homeostasis and blood pressure (BP) through regulation of extracellular water (ECW) volume and arterial tone. Aldosterone excess promotes vascular, renal, and cardiac damage, electrolyte disturbances, and inflammation [5–7]. For the same degree of BP elevation, primary aldosteronism is characterized by worse outcome than essential hypertension, largely because of increased risks of stroke, coronary artery disease, atrial fibrillation, and heart failure [6,7]. Early investigations using radiolabelled tracers in small patient groups indicated that aldosterone excess is characterized by volume overload [8,9]. Recent studies utilizing bioimpedance have confirmed extracellular fluid overload in primary aldosteronism patients [10–12]. Aldosterone excess increases growth factors and collagen accumulation in the arteries [6,7]. Subsequently, many primary aldosteronism patients have higher large artery stiffness than patients with essential hypertension [11]. Targeted treatment of primary aldosteronism has been shown to abolish the excess cardiovascular risk [2,6,13]. However, whether targeted interventions lead to a reversal in ECW volume and arterial tone remains unclear.

The pressure wave generated by left ventricular contraction is reflected backward from the periphery. The forward wave amplitude (FWA), backward wave amplitude (BWA), and the effect of the reflected wave on central SBP (augmentation index, AIx), can be evaluated using pulse wave analysis [11,14]. Despite the finding of higher pulse wave velocity (PWV) in primary aldosteronism, AIx has been similar in primary aldosteronism and essential hypertension [11,14]. Recently, patients with primary aldosteronism were reported to have higher FWA and BWA than patients with essential hypertension, and increased FWA and BWA were suggested to reflect aldosterone-induced vascular damage [14]. However, the effects of volume status on the pressure waves were not addressed [14]. FWA depends crucially on the level of BP [15], and we found no differences in FWA between primary aldosteronism and essential hypertension patients with similar BP values [11].

A wealth of evidence indicates that primary aldosteronism should be detected and treated early in the course of the disease [3,4]. The current primary aldosteronism screening test, ARR, has a sensitivity ranging 22–50%, incorrectly classifying many patients with primary aldosteronism as not having the disease [1,16]. Albeit primary aldosteronism is considered to be volume hypertension [8–11], very few articles have addressed the influence of volume overload on central haemodynamics and wave reflections in primary aldosteronism patients. Here we examined haemodynamic changes induced by targeted treatment of primary aldosteronism in comparison with essential hypertension patients and unmedicated normotensive controls, and tested the hypothesis whether determinations of ECW volume, FWA and BWA can improve diagnostic accuracy and evaluation of treatment efficacy of primary aldosteronism.

## METHODS

### Participants

After screening for secondary hypertension and established primary aldosteronism diagnosis, primary aldosteronism patients from all five University Clinics in Finland are referred to Tampere University Hospital for adrenal vein sampling (AVS) [17,18]. Lateralization in AVS would indicate the need for operative primary aldosteronism treatment [1,19]. The referred primary aldosteronism patients were invited to participate in our study on the haemodynamics of hypertension (Eudra-CT 2006-002065-39, ClinicalTrials.gov NCT01742702). The present analyses comprised 40 primary aldosteronism patients subjected to two separate haemodynamic recordings median 2.8 years apart. The 40 patients with essential hypertension and 40 normotensive controls were enrolled from occupational healthcare providers, personnel and patients treated at Tampere University Hospital, personnel of Tampere University, and clients of Varala Sports Institute during 2006–2019 [11,20,21]. These individuals were selected solely based on their age, sex, BMI, and available data from two separate haemodynamic recordings. The groups were primary aldosteronism, essential hypertension, and unmedicated controls (Table 1).

**TABLE 1:** Demographic and clinical characteristics, blood haemoglobin, and fasting plasma determinations of the participants, with potassium and sodium concentrations also after the follow-up

	Unmedicated controls (*n* = 40)	Essential hypertension (*n* = 40)	Primary aldosteronism (*n* = 40)
Male/female (*n*)	30/10	31/9	30/10
Age (years)	44.1 (8.6)	50.2 (10.4)^∗^	55.2 (9.2)^∗^
Height (cm)	178.3 (9.0)	176.7 (8.6)	177.1 (8.8)
Weight (kg), initial	83.2 (12.6)	89.2 (15.0)	96.0 (19.0)^∗^
Weight (kg), final	82.1 (12.4)	90.9 (15.3)^∗^	97.6 (18.2)^∗^
BMI (kg/m^2^), initial	26.1 (3.2)	28.6 (4.7)^∗^	30.5 (5.1)^∗^
BMI (kg/m^2^), final	25.8 (3.1)	29.1 (4.6)^∗^	31.1 (5.3)^∗^
Type 2 diabetes (*n*)	0	3	12^∗^^,^^†^
Alcohol (standard drinks/week)	4 [0–5]	4 [1–8]	2 [0–5]
Current smokers (*n*)	4	3	6
Office measurements			
SBP (mmHg)	129 (14)	159 (19)^∗^	154 (16)^∗^
DBP (mmHg)	85 (8)	98 (10)^∗^	92 (13)^∗^
Heart rate (bpm)	63 (7)	67 (10)	67 (12)
Blood haemoglobin (g/l)	149 (10)	148 (11)	149 (12)
NT-proBNP (pg/ml)	29.8 [18.2–47.1]	53.6 [34.8–88.8]^∗^	103.6 [61.4–189.8]^∗^^,^^†^
NT-proANP (ng/ml)	2.59 [1.71–3.82]	3.29 [1.93–4.34]	4.70 [3.34–6.60]^∗^^,^^†^
C-reactive protein (mg/l)	0.6 [0.5–1.4]	0.7 [0.5–2.1]	1.2 [0.5–2.2]
Potassium (mmol/l), initial	3.8 (0.3)	3.8 (0.3)	3.5 (0.5)^∗^^,^^†^
Potassium (mmol/l), final	3.8 (0.3)	3.9 (0.3)	4.1 (0.3)^∗^^,^^†^
Sodium (mmol/l), initial	140.4 (1.5)	140.2 (2.5)	142.6 (2.4)^∗^^,^^†^
Sodium (mmol/l), final	140.4 (1.8)	140.6 (2.1)	140.4 (2.2)
Cystatin-C (mg/l)	0.82 (0.14)	0.92 (0.13)^∗^	1.00 (0.18)^∗^^,^^†^
Creatinine (μmol/l)	77 (11)	77 (12)	79 (14)
Total cholesterol (mmol/l)	5.0 (1.0)	5.3 (1.0)	4.6 (1.1)^†^
HDL cholesterol (mmol/l)	1.5 (0.3)	1.4 (0.4)	1.3 (0.5)
LDL cholesterol (mmol/l)	3.0 (0.9)	3.3 (0.9)	3.0 (0.9)
Triglycerides (mmol/l)	1.1 [0.8–1.5]	1.3 [0.9–1.8]	1.4 [0.9–1.8]
Glucose (mmol/l)	5.3 (0.5)	6.1 (1.7)^∗^	6.4 (1.1)^∗^
Insulin (mU/l)	5.7 [4.6–10.2]	10.4 [6.0–16.6]^∗^	14.0 [8.4–18.5]^∗^
QUICKI	0.361 (0.037)	0.338 (0.048)^∗^	0.320 (0.033)^∗^

Missing data: office blood pressure from one patient in the primary aldosteronism group, NT-proBNP concentrations from five unmedicated controls and eight patients with essential hypertension, and final sodium and potassium concentrations from seven unmedicated controls and six patients with essential hypertension. Mean (standard deviation) or median [25th-75th percentile]; NT-proANP and NT-proBNP, N-terminal proatrial and B-type natriuretic peptide, respectively; HDL, high-density lipoprotein; LDL, low-density lipoprotein; QUICKI, quantitative insulin sensitivity check index.

∗ *P* *<* 0.05 vs. unmedicated controls.

† *P* *<* 0.05 vs. essential hypertension.

For collection of background data, exclusion criteria, and primary aldosteronism diagnosis, see Supplemental Methods and Supplemental Table S1. Based on AVS, the primary aldosteronism patients were allocated to adrenalectomy (*n* = 20) or spironolactone-based treatment (*n* = 20) [17]. Among the 40 primary aldosteronism and 40 essential hypertension patients, spironolactone was used by one patient in each group at the first visit, and by 21 and 9 patients, respectively, at the end of the follow-up. Medications are detailed in Supplemental Tables S2 and S3.

### Laboratory analyses

Initially plasma renin activity (PRA) was analysed (DiaSorin radioimmunoassay, Saluggia, Italy), and later direct renin concentration was determined (LIAISON immunoanalyzer, DiaSorin, Saluggia, Italy). With very low values, the results are given as the low detection limits: 0.2 ng/ml/h for PRA, 2 mU/l for renin concentration. Aldosterone was analysed using liquid chromatography–mass spectrometry (LC–MS/MS) as described earlier [22]. For other laboratory analyses, see Supplemental Methods.

### Pulse wave analysis, whole-body impedance cardiography, and experimental protocol

Haemodynamics were recorded in a temperature-controlled laboratory. Pulse wave analysis from the left radial artery (SphygmoCor PWMx, AtCor medical, Australia) was performed as previously described [11,23,24]. Whole-body impedance cardiography (CircMon, JR Medical Ltd., Tallinn, Estonia) was performed to capture heart rate, stroke volume, cardiac output, ECW, and PWV as previously reported [11,24–26]. A summary of these recordings is detailed in Supplemental Methods.

Before the recordings, smoking, caffeine, and heavy meals were to be avoided for at least 4 h, and alcohol for more than 24 h. The participants rested in supine position for ∼10 min, and thereafter supine haemodynamics were recorded for 5 min. The mean values of each 1 min period were calculated for statistics. The follow-up measurements of haemodynamics were performed after a median time of 0.67 and 0.47 years in the unmedicated controls and essential hypertension patients, respectively (Supplemental Table S4). The repeatability and reproducibility of the measurements has been demonstrated [27].

### Statistics

Demographic and laboratory data were analysed using analysis of variance (ANOVA). With skewed distribution, the Kruskal–Wallis test with post hoc Dunn's test were applied. Generalized estimating equations (GEE) was applied to compare haemodynamics during repeated measurements. Linear scale response was applied with the autoregressive option, as successive recordings of hemodynamic variables are autocorrelated. The groups presented with differences in age, cystatin-C, BMI, and number of diabetic patients (Table 1). If these variables correlated with the hemodynamic variable of interest (Pearson *P* < 0.1), they were included in GEE analyses as covariates. The PWV analyses were additionally adjusted for mean aortic pressure [28]. Analyses of haemodynamic changes were adjusted for differences in the follow-up times. Lean body mass instead of BMI was used in analyses of ECW that is more appropriate for normalization of body fluid volumes [29].

Linear regression analysis with stepwise elimination was applied to evaluate the relations between PWV, heart rate, stroke volume, systemic vascular resistance, and ECW volume (independent variables), and FWA and BWA (dependent variables). In BWA analyses, FWA was also included as an independent variable. The changes in the haemodynamic variables were also tested as potential explanatory factors for the changes in FWA and BWA.

The Bonferroni correction was applied in all post hoc analyses. The results were presented as mean and standard error of the mean (SEM), mean and standard deviation (SD), or median (25th to 75th percentile). *P* less than 0.05 was considered significant. SPSS version 28.0 (IBM SPSS Statistics, Armonk, New York, USA) was used.

## RESULTS

### Study population

Ninety-one (76%) male and 29 (24%) female individuals participated in the study (age range 22–71 years) (Table 1). Sex distribution was matched in all groups, whereas normotensive controls were younger (44 years) with lower initial and final BMI (26 kg/m^2^) than participants in the other groups. The number of type 2 diabetic patients was highest among primary aldosteronism patients. Alcohol intake and smoking habits were similar in the groups. Office SBP was ∼25 to 30 mmHg higher, and office DBP was ∼7−13 mmHg higher, in the primary aldosteronism and essential hypertension groups than in normotensive controls (Table 1). The results of screening and confirmatory testing for primary aldosteronism are shown in Supplemental Table S1.

Office heart rate, blood haemoglobin, and plasma concentrations of C-reactive protein, creatinine, high-density lipoprotein (HDL) and low-density lipoprotein (LDL) cholesterol, and triglycerides did not differ in the groups (Table 1). Plasma N-terminal pro-B-type natriuretic peptide (NT-proBNP) was higher in both hypertensive groups than in controls, whereas both NT-proBNP and N-terminal pro-atrial natriuretic peptide (NT-proANP) were highest in the primary aldosteronism group. Plasma total cholesterol was lower in the primary aldosteronism than in essential hypertension group, whereas 16 primary aldosteronism patients but only 6 essential hypertension patients were initially taking statins (Supplemental Table S2). Initial plasma potassium was lowest, whereas plasma sodium concentration was highest, in the primary aldosteronism group. After follow-up, plasma potassium was highest in primary aldosteronism patients without differences in plasma sodium concentration. Fasting plasma glucose and insulin were higher, and insulin sensitivity was lower, in the primary aldosteronism and essential hypertension groups than in controls (Table 1).

Initially, the average number of antihypertensive medications was higher in the primary aldosteronism group than in the essential hypertension group (Supplemental Table S3). At the end of study, the number of antihypertensive medications did not differ between these groups. The defined daily doses (DDD) of antihypertensive agents were higher among the primary aldosteronism patients than in essential hypertension patients during the first visit and at the end of the follow-up (*P* < 0.015). However, in essential hypertension patients, the DDD increased while in primary aldosteronism patients, the DDD decreased during the study. This was attributed to reduced antihypertensive medication use in adrenalectomized patients (Supplemental Table S3).

### Haemodynamics before and after targeted treatment of primary aldosteronism

**FIGURE 1:**
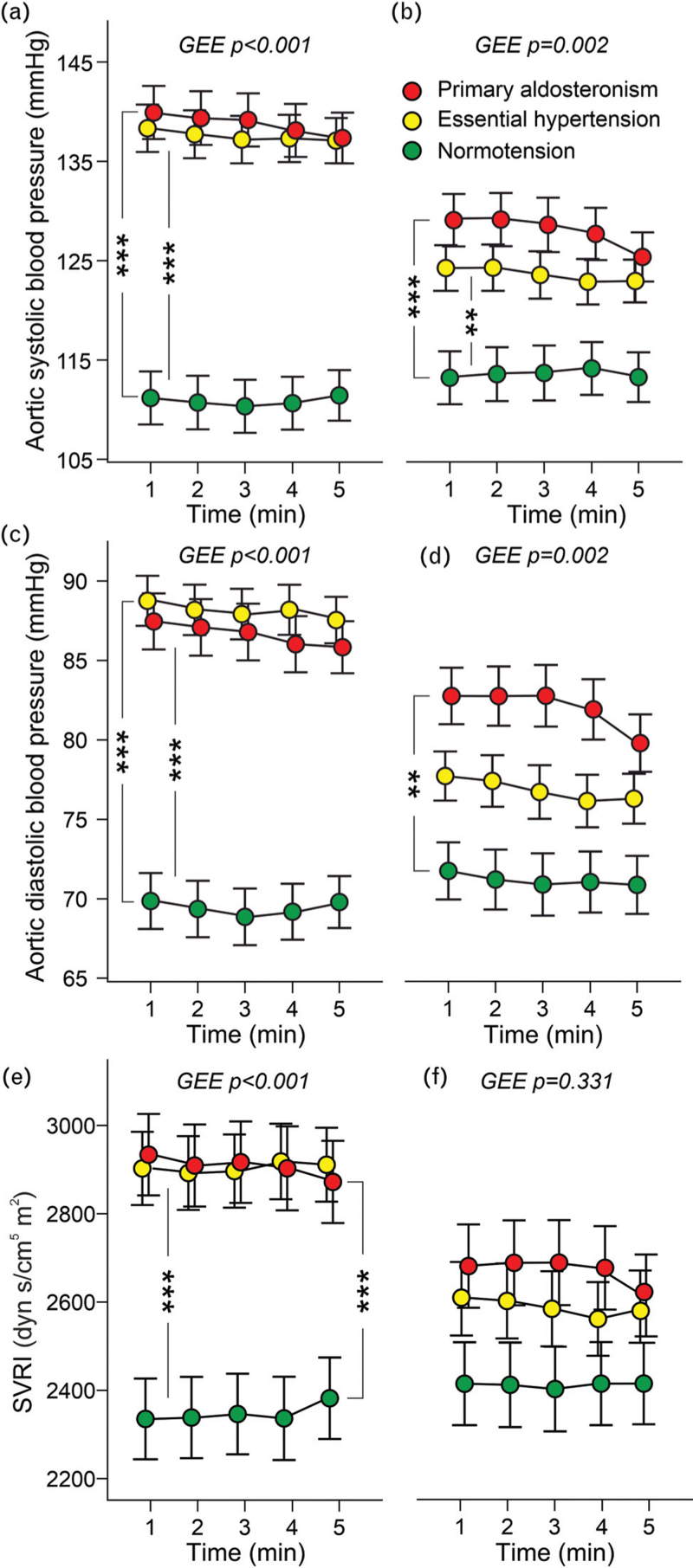
Aortic SBP and DBP and systemic vascular resistance index during 5 min recordings in the beginning of the study (a, c, e, respectively), and at the end of the follow-up (b, d, f, respectively); the analyses were adjusted for age, cystatin-C, presence of diabetes, and BMI, as appropriate; *n* = 40 in each group; GEE, generalized estimating equations, mean and standard error of the mean; ^∗∗^*P* < 0.01, ^∗∗∗^*P* < 0.001.

Initial aortic SBPs and DBPs were similar in the primary aldosteronism and essential hypertension groups and higher than in controls (Fig. 1a and c). The BP values were similarly reduced following treatment in primary aldosteronism and essential hypertension (Supplemental Table S4). However, SBP in both hypertensive groups, and DBP in primary aldosteronism patients, remained higher than in unmedicated controls (Fig. 1b and d). Initial systemic vascular resistance index (SVRI) was similarly elevated in the primary aldosteronism and essential hypertension groups (Fig. 1e), while at the end of the follow-up, SVRI was not significantly different between the study groups (Fig. 1f). The treatment-induced reductions in SVRI did not differ in the primary aldosteronism and essential hypertension groups (Supplemental Table S4).

**FIGURE 2:**
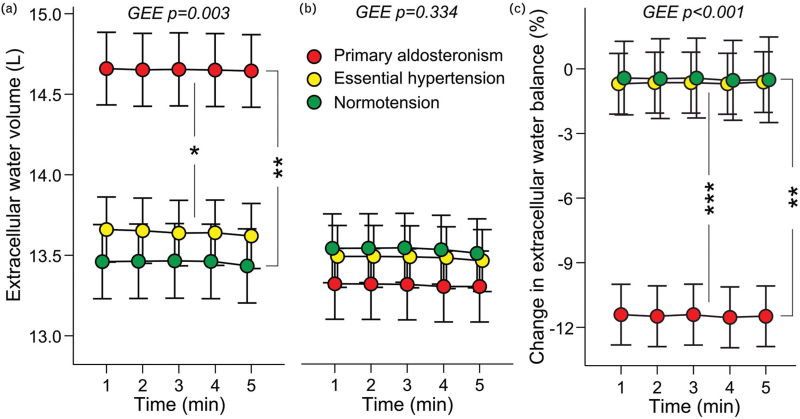
Extracellular water volume in the beginning (a) and at the end of the follow-up (b) adjusted for age, cystatin-C, presence of diabetes, and estimated lean body mass; and change in extracellular water balance during the study (c), *n* = 38–40 in each group; GEE, generalized estimating equations, mean and standard error of the mean; ^∗^*P* < 0.05, ^∗∗^*P* < 0.01, ^∗∗∗^*P* < 0.001.

Initially the primary aldosteronism patients presented with ∼1 l (10%) excess of ECW volume versus the other groups (Fig. 2a). Following treatment the primary aldosteronism patients presented with a significant reduction in ECW balance, and their excess ECW volume was normalized (Fig. 2b and c, Supplemental Table S4). This change was observed in the absence of a significant reduction in body weight in the primary aldosteronism patients (Table 1). Of note, in bioimpedance measurements the ECW volume comprises ∼40% of total body water [30].

**FIGURE 3:**
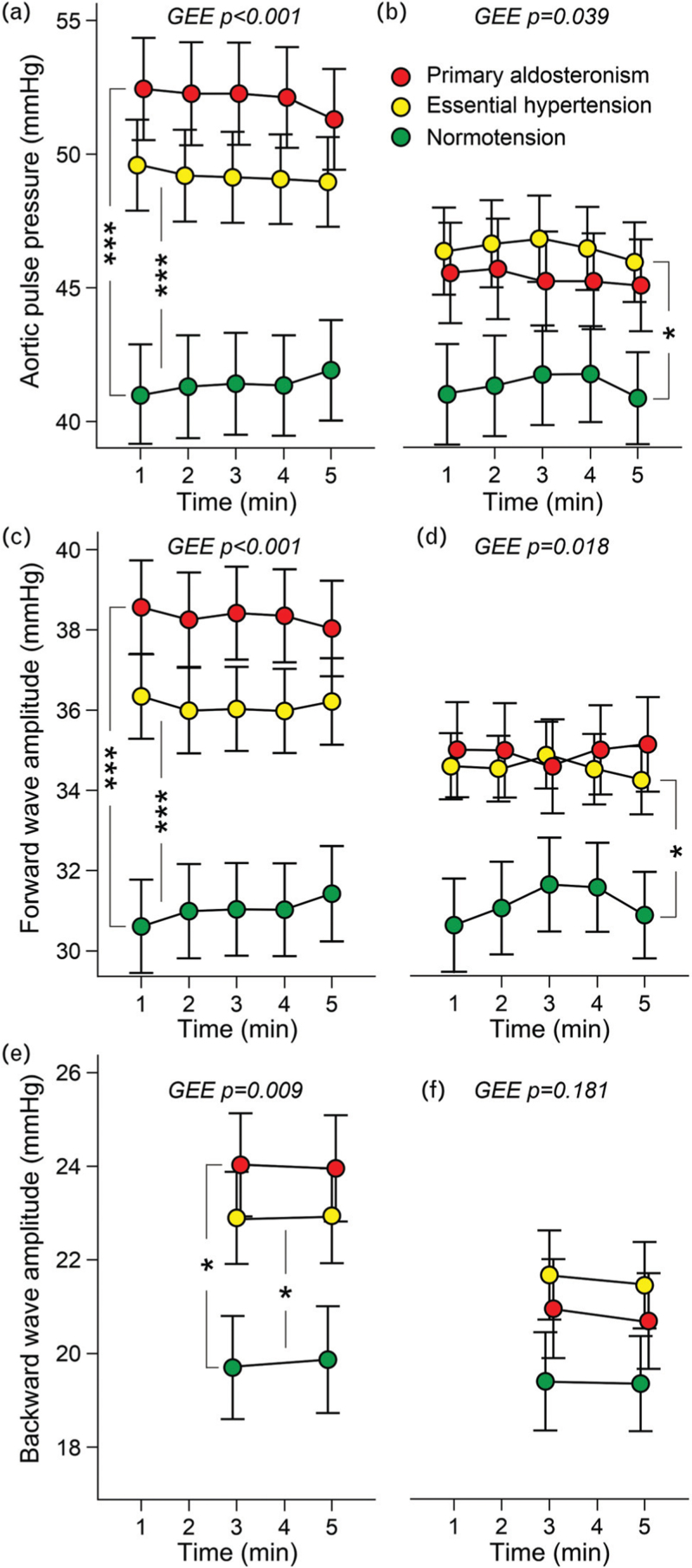
Aortic pulse pressure in the beginning (a) and at the end of the follow-up (b); and respective results of forward wave amplitude (c and d), and backward wave amplitude (e and f) adjusted for age, cystatin-C, presence of diabetes, BMI; *n* = 40 in each group; GEE, generalized estimating equations, mean and standard error of the mean; ^∗^*P* < 0.05, ^∗∗∗^*P* < 0.001.

Before the treatments, aortic pulse pressure, FWA, and BWA were higher in both hypertensive groups than in controls (Fig. 3a, c, e). Following treatment, the changes in aortic pulse pressure and FWA did not differ in the primary aldosteronism and essential hypertension groups, whereas the changes in these variables were more pronounced in primary aldosteronism patients than in untreated controls (Supplemental Table S4). In the essential hypertension group, aortic pulse pressure and FWA remained higher than in unmedicated controls (Fig. 3b and d). Albeit the numerical reductions in BWA did not deviate between the groups (Supplemental Table S4), the BWAs were no longer different in the groups at close of the study (Fig. 3f).

Heart rate and cardiac output related to body surface area (cardiac index) did not differ between the groups before or after the treatments (Supplemental Figures S1A-D). Before the treatments, unadjusted aortic-to-popliteal PWV was higher in primary aldosteronism and essential hypertension than in controls (Supplemental Figure S2A). Following treatment, the unadjusted PWV remained highest in the primary aldosteronism group (Supplemental Figure S2B). However, when adjusted for demographics, metabolic factors, and mean aortic BP [28], PWV was corresponding in all groups before and after the treatments (Supplemental Figures S2C-D). The treatment-induced change in PWV was higher in essential hypertension patients than in normotensive controls (Supplemental Table S4).

#### Adrenalectomy vs. medical treatment of primary aldosteronism

None of the haemodynamic variables differed in the initial levels, or in the treatment-induced changes, when the adrenalectomized primary aldosteronism patients and medically treated primary aldosteronism patients were compared (data not shown).

### Explanatory factors for forward and backward wave amplitudes in regression analyses

To examine the relationships of FWA and BWA with haemodynamic factors linear regression analyses were performed (Table 2). Systemic vascular resistance, stroke volume, heart rate, and ECW volume were explanatory factors for FWA. Changes in systemic vascular resistance and ECW volume were explanatory factors for the change in FWA. When the presence of primary aldosteronism, essential hypertension, type 2 diabetes, demographic factors, and results of laboratory determinations were included in the analyses, the explanatory factors for FWA were the presence of primary aldosteronism and essential hypertension, and body weight (Supplemental Table S5).

**TABLE 2:** Forward and backward wave amplitudes^a^

Forward wave amplitude	*B*	Beta	*P*
*R*^2^ = 0.332			
Constant	−39.364		<0.001
Extracellular water volume	0.790	0.220	0.015
Systemic vascular resistance	0.016	0.678	<0.001
Stroke volume	0.226	0.523	<0.001
Heart rate	0.302	0.391	<0.001

Change in forward wave amplitude	*B*	Beta	*P*
*R*^2^ = 0.162			
Constant	−0.686		0.256
Change in systemic vascular resistance	0.008	0.380	<0.001
Change in extracellular water volume	0.931	0.183	0.038

Backward wave amplitude	*B*	Beta	*P*
*R*^2^ = 0.587			
Constant	−1.661		0.729
Forward wave amplitude	0.452	0.467	<0.001
Systemic vascular resistance	0.006	0.266	<0.001
Pulse wave velocity	1.183	0.278	<0.001
Heart rate	−0.159	−0.212	0.004

Change in backward wave amplitude	*B*	Beta	*P*
*R*^2^ = 0.643			
Constant	−0.008		0.984
Change in forward wave amplitude	0.588	0.599	<0.001
Change in heart rate	−0.295	−0.427	<0.001
Change in pulse wave velocity	1.470	0.252	<0.001

a Linear regression analyses with stepwise elimination; pulse wave velocity, heart rate, stroke volume, systemic vascular resistance, and extracellular water volume included as potential explanatory factors for FWA; in addition, FWA was included as a potential explanatory for BWA. The changes in the above variables were tested as potential explanatory factors for the changes in FWA and BWA. B, unstandardized coefficient; Beta, standardized coefficient.

The explanatory factors for BWA were FWA, systemic vascular resistance, PWV, and heart rate (Table 2). The explanatory factors for the treatment-induced change in BWA were changes in FWA, heart rate, and PWV. The *R*^2^ values for the analyses concerning BWA were clearly higher than the *R*^2^ values for the analyses concerning the FWA (Table 2).

## DISCUSSION

Relatively few studies have assessed haemodynamic changes after targeted treatment of primary aldosteronism. The present results indicate that primary aldosteronism patients had increased ECW volume, and that excess volume load elevated FWA. Prior to the treatments, SVRI was similarly elevated in primary aldosteronism and essential hypertension patients, and following the treatments, SVRI was no longer different from control in either group. Although a previous study suggested that increased FWA and BWA indicate aldosterone-induced vascular damage [14], our results show similar elevations of these variables in primary aldosteronism and essential hypertension, with corresponding treatment-induced reductions. Regression analyses indicated that ECW volume directly influenced FWA and indirectly affected BWA through its impact on FWA. The targeted treatments normalized volume status in primary aldosteronism.

The median follow-up times were not equal in the groups and ranged 0.47–2.80 years. However, the emphasis was on repeated haemodynamic recordings to ensure similar level of habituation during the recordings. Importantly, no change in the ECW status was observed in the essential hypertension group and untreated controls, and all analyses of the haemodynamic changes were adjusted for the differences in follow-up time, in addition to adjustments for age, cystatin-C, presence of diabetes, and BMI.

The primary aldosteronism group presented with typical characteristics of aldosterone excess: elevated ARR and 24 h aldosterone excretion, and lower plasma potassium and higher sodium than patients with essential hypertension [1,11]. These electrolyte deviations were corrected by treatment of primary aldosteronism. The patients with primary aldosteronism and essential hypertension presented with similar elevations of BP whether measured in the office or in the laboratory. After the follow-up, the changes in BP and haemodynamic variables were consistent in the primary aldosteronism and essential hypertension groups, except for the reduction in ECW volume, which was observed only in primary aldosteronism.

In unilateral primary aldosteronism, adrenalectomy is the most effective treatment [31]. When compared with surgical treatment, primary aldosteronism patients treated with mineralocorticoid receptor antagonists require higher doses of antihypertensive agents [32]. In our adrenalectomized primary aldosteronism patients, the number and DDD of antihypertensive medications decreased, and potassium supplementation was discontinued, aligning with prior findings [33–35]. Except for one individual, all patients in the medically treated primary aldosteronism group received spironolactone at the end of the study. During aldosterone excess, BP lowering by spironolactone has been related to its diuretic action, whereas in the absence of aldosterone excess, the antihypertensive action is mainly attributed to reduced systemic vascular resistance [13].

The role of blood volume as a regulator of BP may have been underestimated [36]. When ECW volume increases, it increases plasma volume, and elevates BP [36]. Volume status and BP regulation are intimately correlated [37]. Earlier reports have shown overhydration and increased ECW volume in primary aldosteronism vs. essential hypertension [8–12], whereas BP reduction has been reported to correlate with plasma volume contraction in primary aldosteronism [38]. We observed ∼1 l ECW excess in the primary aldosteronism patients, which was normalized by adrenalectomy and spironolactone-based treatment. The current findings propose that incorporating ECW evaluation alongside routine diagnostics could enhance the identification and diagnosis of primary aldosteronism. Measurement of volume status using bioimpedance spectroscopy is a quick and commonly used method for measuring volume status in patients with chronic renal failure [39]. Increased systemic vascular resistance in the absence of changes in cardiac output is the typical characteristic in patients with essential hypertension [40]. This corresponds to the present essential hypertension group, as the reduction of BP following treatment of essential hypertension was explained by reduced SVRI.

ANP concentration is higher in atrial than in ventricular tissue. In response to cardiomyocyte stretch, ANP is released into the circulation from secretory granules [41]. BNP is synthetized in atrial and ventricular cardiomyocytes, but BNP is not stored to the same degree as ANP [41]. Thus, ANP more acutely responds to increases in volume load, whereas elevated BNP more indicates pressure and volume overload upon the heart [41]. In the present study, initial plasma NT-proANP concentration was highest in the primary aldosteronism group, indicating increased ECW volume. In contrast, plasma NT-proBNP was increased in essential hypertension and primary aldosteronism patients, with a 1.4-fold elevation in essential hypertension in response to pressure overload, and a 3.5-fold elevation in the primary aldosteronism group in response to volume and pressure overloads. Higher initial NT-proANP concentration and ECW volume were not related with increased cardiac output in primary aldosteronism. At steady state, venous return equals cardiac output. However, venous return, which refers to the inflow to the right heart, is influenced not only by the current central venous pressure distending the right atrium but also by venous resistance to flow [42]. Although higher ECW volume and elevated NT-proANP concentration in the current primary aldosteronism group imply increased right atrial distension, the resistance to venous flow in these patients remains undisclosed. Additionally, the absence of a rise in cardiac output among primary aldosteronism patients may be partly attributed to the higher prevalence of beta adrenoceptor blocker usage in the primary aldosteronism group (65%) compared with the essential hypertension group (22%).

Several studies have reported increased large arterial stiffness in primary aldosteronism patients [11,43–45]. A meta-analysis suggested that mineralocorticoid receptor antagonist (MRA) treatment can reduce PWV independent of the lowering of BP in primary aldosteronism [46]. In the present study, initial unadjusted PWV was higher in patients with primary aldosteronism and essential hypertension than in normotensive controls. After follow-up, PWV remained highest in primary aldosteronism patients, whereas in essential hypertension patients, PWV did not deviate from controls. However, after adjustment for confounders, none of the PWV differences remained significant. Thus, the observed differences in PWV were more explained by differences in age, mean central BP, renal function, adiposity, and impaired glucose metabolism than by aldosterone excess itself. Still, the above adjustments do not eliminate the fact that PWV *in vivo* remained highest in the primary aldosteronism group. This finding may also explain why aortic SBP remained elevated in the primary aldosteronism group.

The FWA is mainly determined by left ventricular ejection, aortic stiffness, and aortic diameter, and it is a major determinant of central pulse pressure. The level of the BWA is influenced by FWA and also by large artery stiffness [44]. In the present study, FWA and BWA in primary aldosteronism patients were not different from those in essential hypertension patients before or after the treatments. In regression analyses, ECW volume was an explanatory factor for FWA, whereas the explanatory factors for treatment-induced reduction in FWA were decreased SVRI and reduced ECW volume. The explanatory factors for reduced BWA after treatment were changes in FWA, heart rate, and PWV. Taken together, volume excess in primary aldosteronism directly elevated FWA, and indirectly elevated BWA via its effect on FWA.

The present study has limitations. The participant number was rather small, and therefore we matched especially the patients in the essential hypertension and primary aldosteronism groups. The unmedicated controls were younger than the hypertensive patients, the follow-up times were shorter in the unmedicated and essential hypertension groups, whereas the proportions of type 2 diabetic patients were higher in the primary aldosteronism group. To control confounding, the analyses were adjusted for these differences. The variables ECW volume, stroke volume, and cardiac output were mathematically evaluated from the bioimpedance signal, which simplifies physiology [25]. Nevertheless, the above methods have been validated in comparisons with invasive measurements, three-dimensional ultrasound, and tonometric PWV recordings [24–26]. Central BP and pressure waves were also mathematically derived from the radial artery tonometric signal [23]. Criticism regarding the reliability of the tonometric BP recording has been presented [47], but we found that tonometric BP values in the laboratory well corresponded to ambulatory daytime BP [21].

In conclusion, FWA, BWA, and systemic vascular resistance exhibited similar elevation and reduction through treatment in patients with primary aldosteronism and essential hypertension. In contrast, primary aldosteronism patients presented with an excess volume load that was corrected by treatment. The increased ECW volume also contributed to elevated FWA and indirectly affected BWA via its influence on FWA. Our results underscore the importance of considering ECW volume when assessing the cardiovascular impact and treatment efficacy in aldosterone excess.

## ACKNOWLEDGEMENTS

The authors express gratitude to research nurses Emmi Hirvelä, Virpi Ryhänen, and Paula Liikanen. The CSC – IT Center for Science, Finland, is acknowledged for computational resources.

Sources of funding: State Funding for University-Level Health Research in Tampere University Hospital, Wellbeing Services County of Pirkanmaa (9T052, 9X046, 9AA062); Finnish Foundation for Cardiovascular Research, Sigrid Jusélius Foundation, Pirkanmaa Regional Fund of the Finnish Cultural Foundation, Aarne Koskelo Foundation, Ida Montin Foundation, Päivikki and Sakari Sohlberg Foundation, and Helsinki University Hospital research grants (TYH2022311).

The work presented in the article has not been presented previously in whole or in part.

Data availability statement: the datasets of the study contain several indirect identifiers and are not available publicly. The datasets are available from the corresponding author on reasonable request.

### Conflicts of interest

There are no conflicts of interest.

## Supplementary Material

Supplemental Digital Content
