# Supplementary material for: Volume overload is a major characteristic in primary aldosteronism: a 3-year follow-up study
Source: J Hypertens. 2024 Feb 21;42(6):1057–65. doi: 10.1097/HJH.0000000000003696 (PMC11064919; doi:10.1097/HJH.0000000000003696)
Supplement: Supplemental Digital Content [file jhype-42-1057-s001.pdf]

Volume overload is a major characteristic in primary aldosteronism: a 3-year follow-up study

Eeva Kokko[1*], Manoj Kumar Choudhary[1*], Aapo Mutanen[1], Milja Honkonen[1], Antti Tikkakoski[1,2], Jenni K. Koskela[1,3], Mari Hämäläinen[1,4], Eeva Moilanen[1,4], Marianna Viukari[5], Niina Matikainen[5], Pasi I. Nevalainen[3], Ilkka Pörsti[1,3]

Short title: Aldosteronism and extracellular water volume

[*]Equal contribution, joint first authors.

[1]Faculty of Medicine and Health Technology, Tampere University, Tampere;

[2]Department of Clinical Physiology and Nuclear Medicine, Tampere University Hospital, Tampere;

[3]Department of Internal Medicine, Tampere University Hospital, Tampere;

[4]Immunopharmacology Research Group, Tampere University and Tampere University Hospital, Tampere;

[5]Endocrinology, Helsinki University Hospital and Research Programs Unit, Clinical and Molecular Medicine, University of Helsinki, Helsinki, Finland.

Corresponding author:

Ilkka Pörsti, MD

Faculty of Medicine and Health Technology

Tampere University

Tampere, Finland;

Tel: +358 50 4389440

ORCID ID 0000-0002-4905-7564

E-mail: ilkka.porsti@tuni.fi

1. Supplemental Methods

1.1. Background data, exclusion criteria, and diagnosis of primary aldosteronism

Office blood pressure (BP) measurements and laboratory analyses for elevated BP were performed according to guidelines [1]. A physician examined the participants, and medical history, lifestyle habits, medicines, smoking status, and alcohol consumption as standard drinks pr week (~12 grams of absolute alcohol) were documented.

Exclusion criteria were coronary artery disease, stroke, heart failure, valvular heart disease, chronic kidney disease, secondary hypertension other than primary aldosteronism (PA), alcohol or substance abuse, psychiatric illness other than mild depression or anxiety, and heart rhythm other than sinus rhythm. The study complies with the declaration of Helsinki and was approved by the ethics committee of the Tampere University Hospital (code R06086M). Signed informed consent was obtained from all participants.

PA diagnosis was based on screening and confirmatory testing [2–5]. Positive screening for aldosteronism (n=40) was defined as serum aldosterone (pmol/l) to plasma renin activity (ng/ml/h) ratio >750, with serum aldosterone ≥280 pmol/l [2,4]; or serum aldosterone (pmol/l) to plasma renin concentration (mU/l) ratio >30, with serum aldosterone ≥280 pmol/l [6]. Fourteen patients presented with hypokalaemia. Confirmatory testing was performed in 39 patients, showing urine aldosterone >33 nmol/day during oral sodium loading (Supplemental Table S1) [2].

1.2. Laboratory analyses

Blood and urine samples were taken after ~12-hours of fasting. Plasma and urine electrolytes, and plasma glucose, cystatin-C, lipids, C-reactive protein, uric acid, and creatinine were determined using Cobas 6000, module c501 (Roche Diagnostics, Basel, Switzerland), and blood cell count by ADVIA 120 or 2120 (Bayer Health Care, Tarrytown, NY, USA). Quantitative insulin sensitivity check index was calculated [7]. N-terminal pro-B-type natriuretic peptide (NT-proBNP) and N-terminal pro-atrial natriuretic peptide (NT-proANP) were determined using enzyme-linked immunosorbent assays (NT-

proBNP ELISA, Abcam, Cambridge, UK; NT-ProANP DuoSet ELISA, R&D Systems Ltd, Abingdon, UK; interassay coefficients of variation 7.5% and 7.2%, respectively). Exclusion of kidney diseases was based on urine dipstick refractometry (Siemens Clinitec Atlas or Advantus, Siemens Healthcare GmbH, Erlangen, Germany) and plasma creatinine and cystatin-C concentrations.

1.3. Pulse wave analysis

Using an automated tonometric sensor (Colin BP-508T, Colin Medical Instruments Corp., USA), BP and pulse wave were captured from the left radial artery kept at heart level. The radial signal was calibrated twice during each 5-minute period by contralateral brachial BP measurements. Aortic BP, AIx (augmented pressure/pulse pressure*100), and AIx adjusted to heart rate 75 beats per minute (AIx@75) were determined (SphygmoCor PWMx®, AtCor medical, Australia) [8].

BWA could not be analysed using our SphygmoCor-2000 software, but waveforms from each minute were stored and analysed using SphygmoCor 9.0 software (AtCor medical) [9]. Two stable time points from the 3rd and 5th minutes of the recordings were chosen for FWA and BWA determinations. The mean FWA of the original 5-minute recordings and the FWA analyses from these two time-points correlated strongly ($r=0.946$, $p<0.001$). Therefore, the BWAs of the selected time-points well represent the 5-minute recordings.

1.4. Whole-body impedance cardiography

Heart rate, stroke volume, cardiac output, extracellular water (ECW), and pulse wave velocity (PWV) were recorded using whole-body impedance cardiography (CircMon®, JR Medical Ltd., Tallinn, Estonia) as previously reported [10]. Systemic vascular resistance was calculated from cardiac output and tonometric BP: assumed normal central venous pressure (4 mmHg) was subtracted from mean arterial pressure and divided by cardiac output. Systemic vascular resistance, stroke volume and cardiac output were related to body surface area (cardiac index, stroke index and systemic vascular resistance index (SVRI), respectively). The stroke volume values measured using CircMon® correspond to measurements using 3 dimensional ultrasound [11], and cardiac output corresponds to

values of thermodilution (bias 0.00 l/min, 95% confidence interval -0.26 to 0.26) and Fick direct oxygen methods (bias -0.32 l/min, 95% CI -0.69 to 0.05) [10].

The CircMon® evaluates ECW volume by the formula ECW = k*($Height^2$/Z). The coefficient k (Ω*cm) is derived from blood resistivity and distance of voltage electrodes, height is given in cm, and Z is the recorded body impedance. The bioimpedance-derived ECW volume correlates well with ^{51}Cr-EDTA dilution based ECW measurement (n=15, r=0.74, bias 0.2±1.1 l, mean±SD) [12]. The calculation of ECW balance has been described previously [3].

For PWV analysis, the CircMon® records the time difference between the onset of the decrease in the impedance of the whole-body and popliteal artery signal [13]. PWV is calculated from the time difference and the electrode distance. This method slightly overestimates PWV, whereby a validated equation was applied to calculate values that agree with ultrasound method (PWV = $PWV_{impedance}$ * 0.696 + 0.864) [13]. The recorded PWV values correlate well with values obtained using SphygmoCor® (r=0.82, bias 0.02 m/s, 95% CI -0.21 to 0.25) [14] and ultrasound (r=0.91) [13].

1.6. Sample size and missing values

Sample size was based on the assumption of ~5% (8%) [mean (standard deviation, SD)] ECW excess in PA [3,15]. Power analysis indicated that ≥39 subjects per group were required (alpha 0.05, power 80%).

Missing values: Aortic systolic BP from minutes 3 and 4 of the final recording were missing from one control, and the missing BP and other haemodynamic values requiring information about systolic BP were replaced by the mean values of the respective variables recorded at minutes 2 and 5. Initial ECW volume was missing from two controls and final ECW volumes from one PA and one EH patient. Initial PWV data was missing form one unmedicated control and final PWV from one PA patient. Missing laboratory values are listed in Table 1 footnote.

Supplemental Table S1. Laboratory characteristics of 40 patients with primary aldosteronism referred to Tampere University Hospital for adrenal vein sampling.

	Median	25th percentile	75th percentile	Number*	Normal range
Lowest plasma potassium (mmol/l)	3.1	2.9	3.4	40	3.3-4.8
Serum aldosterone (pmol/l)	720	569	1088	40	<520
Plasma renin activity (ng of Ang I/ml/h)	0.2	0.2	0.3	29	1.5-5.7
Plasma renin concentration (mU/l)	7.6	5.0	13.0	11	4.4–46
Ratio of aldosterone to renin activity	2810	1713	4370	29	<750
Ratio of aldosterone to renin concentration	67	35	120	11	<30
Urinary aldosterone (nmol/24h)	60.0	51.0	78.0	39	<40
Urinary sodium (mmol/24h)	232	173	268	37	130-240
Urinary potassium (mmol/24h)	114	85	145	34	60-90

*Number of subjects with available result

Supplemental Table S2. Number of subjects using potassium supplements, anti-hypertensive medications, and other medications in the essential hypertension group and primary aldosteronism group during the first visit and at the end of the follow-up.

	**Essential hypertension**	**Primary aldosteronism**		
	(n=40)	All patients (n=40)	Surgical treatment (n=20)	Medical treatment (n=20)
	**First visit / End of follow-up**			
ACE inhibitor	5 / 9	10 / 8	5 / 4	5 / 4
Angiotensin II receptor blocker	6 / 9	21 / 25	12 / 6	9 / 9
Beta blocker	8 / 27	21 / 18	9 / 8	12 / 10
Beta and alpha blocker	1 / 1	5 / 2	4 / 2	1 / 0
Calcium channel blocker	10 / 19	36 / 29	18 / 13	18 / 16
Thiazide	7 / 8	6 / 5	2 / 2	4 / 3
Furosemide	1 / 0	3 / 4	1 / 1	2 / 3
Spironolactone	1 / 9	1 / 21	1 / 2	0 / 19
Moxonidine	1 / 1	6 / 1	4 / 0	2 / 1
Minoxidil	0 / 0	1 / 0	1 / 0	0 / 0
Prazosin	2 / 0	14 / 5	5 / 1	19 / 4
Potassium supplement	1 / 0	24 / 1	14 / 0	10 / 1
Statin	6 / 6	16 / 17	7 / 7	9 / 10
Ezetimib	1 / 0	0 / 0	0 / 0	0 / 0
Acetylsalicylic acid	1 / 3	11 / 12	6 / 7	5 / 5
Metformin	1 / 1	9 / 9	4 / 4	5 / 5
Incretin mimetics	0 / 0	1 / 2	1 / 2	0 / 0
Dipeptidyl-peptidase 4 inhibitor	2 / 2	4 / 5	2 / 2	2 / 3
Sulfonylureas	0 / 0	1 / 0	1 / 0	0 / 0

Insulin	1 / 0	4 / 0	3 / 0	1 / 0
Antihistamines	1 / 1	2 / 2	1 / 1	1 / 1
5α-reductase inhibitors	0 / 0	1 / 0	1 / 0	0 / 0
Warfarin	0 / 0	2 / 2	0 / 0	2 / 2
Allopurinol	0 / 0	1 / 1	0 / 1	0 / 0
Benzodiazepines	1 / 0	1 / 2	1 / 1	0 / 1
Selective serotonin reuptake inhibitors	3 / 2	4 / 1	3 / 1	1 / 0
Other antidepressant	1 / 1	1 / 2	1 / 1	0 / 1
Antiepileptics	0 / 0	1 / 2	0 / 1	1 / 1
Non-steroidal anti-inflammatory drugs	0 / 0	0 / 2	1 / 0	1 / 0
Coxibs	0 / 0	1 / 0	1 / 0	0 / 0
Antiarrhythmic agents	0 / 0	1 / 1	1 / 1	0 / 0
Vitamin D supplement	2 / 2	5 / 5	3 / 2	2 / 3
Calcium supplement	1 / 0	3 / 4	2 / 2	1 / 2
Proton pump inhibitor	1 / 2	1 / 1	1 / 0	0 / 1
Thyroxin	3 / 1	1 / 2	0 / 2	1 / 0

Supplemental Table S3. Number and defined daily doses of antihypertensive agents in the essential hypertension group and the primary aldosteronism group during the first visit and at the end of the follow-up.

	**Essential hypertension** (n=40)	**Primary aldosteronism**		
		All patients (n=40)	Surgical treatment (n=20)	Medical treatment (n=20)
Number of antihypertensive agents				
First visit	1.1 (1.4)	3.1 (1.4)*	3.0 (1.3)	3.1 (1.6)
End of follow-up	2.1 (1.4)†††	2.7 (1.7)	2.0 (1.6)††	3.5 (1.5)
Defined daily doses				
First visit	1.3 (1.9)	4.9 (2.5)*	5.1 (2.2)	4.6 (2.8)
End of follow-up	2.2 (2.2)†††	3.4 (2.6)*††	2.7 (2.5)††	4.1 (2.5)

Mean (SD); for defined daily doses (DDD), see www.who.int/tools/atc-ddd-toolkit/about-ddd;
*p<0.05 vs. essential hypertension; ††p<0.01, †††p<0.001 vs. 1st visit.

Supplemental Table S4. Changes in the haemodynamic variables in the study groups during follow-up.

	Unmedicated controls (n=40)	Essential hypertension (n=40)	Primary aldosteronism (n=40)	P-value
Follow-up time (years)	0.67 [0.38-0.75]	0.47 [0.17-1.15]	2.80 [2.40-4.15]*†	<0.001
Change during follow-up in				
Aortic systolic blood pressure (mmHg)	1.4 (3.3)	-16.1 (3.5)*	-18.7 (4.0)*	<0.001
Aortic diastolic blood pressure (mmHg)	1.5 (2.1)	-12.5 (2.2)*	-9.9 (2.2)*	<0.001
Extracellular water volume (L)	-0.17 (0.23)	-0.07 (0.16)	-1.16 (0.25)*†	0.004
Heart rate (beats/min)	3.5 (1.7)	-2.5 (1.6)*	1.9 (1.5)	0.002
Cardiac index (L/min/m^2)	0.15 (0.11)	0.05 (0.09)	-0.04 (0.08)	0.425
Systemic vascular resistance index (dyn s/cm^5 m^2)	-70 (118)	-434 (96)*	-339 (95)	0.012
Aortic pulse pressure (mmHg)	-0.3 (2.3)	-3.7 (2.2)	-8.5 (2.7)*	0.04
Forward wave amplitude (mmHg)	0.2 (1.4)	-2.1 (1.2)	-4.1 (1.4)*	0.043
Backward wave amplitude (mmHg)	-0.7 (1.4)	-1.9 (1.4)	-5.0 (1.8)	0.100
Pulse wave velocity (m/s)	-0.08 (0.19)	-0.82 (0.20)*	-0.58 (0.17)	0.001

Median [25th-75th percentile] or mean (standard error of the mean); Kruskal-Wallis test and Mann-Whitney U-test for follow-up time; generalized estimating equations for the changes in haemodynamic variables with adjustments for age, body mass index (estimated lean body mass for extracellular water volume), cystatin-C, follow-up time, and presence of type 2 diabetes (and change in mean aortic pressure for pulse wave velocity); *P<0.05 vs. unmedicated controls, †P<0.05 vs. essential hypertension.

Supplemental Table S5. Forward wave amplitude: linear regression analyses with stepwise elimination with age, sex, height, weight, cystatin C concentration, pulse wave velocity, heart rate, stroke volume, systemic vascular resistance, extracellular water volume, presence of primary aldosteronism, presence of essential hypertension, and presence type 2 diabetes as explanatory factors.

**Forward wave amplitude (m / s)**	**B**	**Beta**	***P***
R^2=0.324			
(constant)	16.448		<0.001
Primary aldosteronism	6.423	0.422	<0.001
Essential hypertension	4.443	0.292	0.003
Weight	0.082	0.188	0.029
Pulse wave velocity	0.728	0.165	0.071

Supplemental Figure S1. Heart rate in the beginning (A) and at the end of the follow-up (B) adjusted for age, cystatin-C, presence of diabetes, and body mass index; and respective results for cardiac index (C, D) adjusted for age, cystatin-C, and presence of diabetes; n=40 in each group; GEE, generalized estimating equations, mean and standard error of the mean; ****p*<0.001.

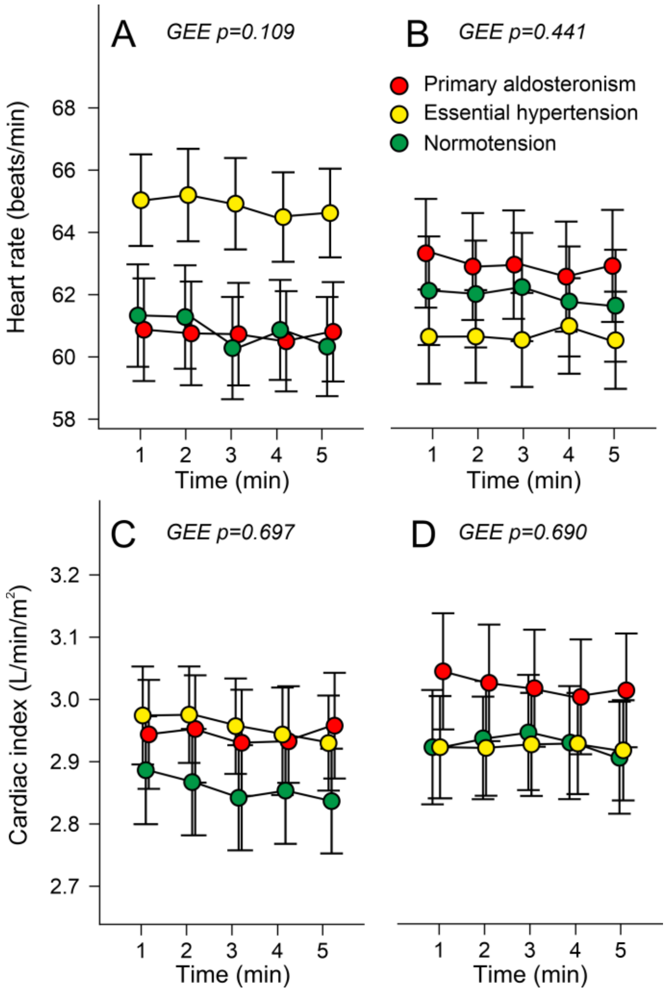

Supplemental Figure S2. Unadjusted pulse wave velocity in the beginning (A) and at the end of the follow-up (B); and pulse wave velocity adjusted for age, cystatin-C, presence of diabetes, body mass index, and mean aortic blood pressure in the beginning (C) and at the end of the follow-up (D); n=39-40 in each group; GEE, generalized estimating equations, mean and standard error of the mean; **p*<0.05, ****p*<0.001.

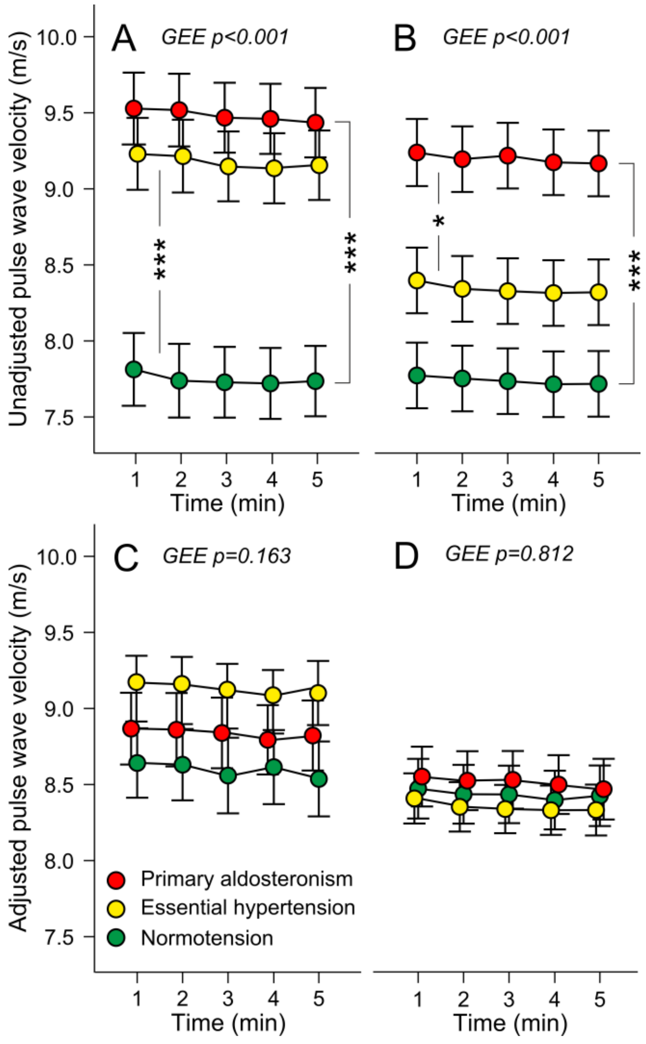

References of the Supplemental Digital Content

1 Williams B, Mancia G, Spiering W, Rosei EA, Coca A, Clement DL, *et al.* 2018 ESC/ESH Guidelines for the management of arterial hypertension. *Eur Heart J* 2018; 39:3021–104.

2 Funder JW, Carey RM, Mantero F, Murad MH, Reincke M, Shibata H, *et al.* The management of primary aldosteronism: case detection, diagnosis, and treatment: an endocrine society clinical practice guideline. *J Clin Endocrinol Metab* 2016; 101:1889–1916.

3 Choudhary MK, Värri E, Matikainen N, Koskela J, Tikkakoski AJ, Kähönen M, *et al.* Primary aldosteronism: Higher volume load, cardiac output and arterial stiffness than in essential hypertension. *J Intern Med* 2021; 289:29–41.

4 Young WF. Diagnosis and treatment of primary aldosteronism: practical clinical perspectives. *J Intern Med* 2019; 285:126–148.

5 Ylänen A, Pörsti I, Nevalainen R, Hinkka T, Huhtala H, Matikainen N, *et al.* Non-suppression of renin by renal cysts in a subset of patients with primary aldosteronism-a prospective observational single center study. *Eur J Endocrinol* 2023; 188:621–629.

6 Ma L, Song Y, Mei M, He W, Hu J, Cheng Q, *et al.* Age-related cutoffs of plasma aldosterone/renin concentration for primary aldosteronism screening. *Int J Endocrinol* 2018; 2018:8647026.

7 Katz A, Nambi SS, Mather K, Baron AD, Follmann DA, Sullivan G, *et al.* Quantitative insulin sensitivity check index: a simple, accurate method for assessing insulin sensitivity in humans. *J Clin Endocrinol Metab* 2000; 85:2402–2410.

8 Chen C-H, Nevo E, Fetics B, Pak PH, Yin FCP, Maughan WL, *et al.* Estimation of central aortic pressure waveform by mathematical transformation of radial tonometry pressure: validation of generalized transfer function. *Circulation* 1997; 95:1827–1836.

9 Westerhof BE, Guelen I, Westerhof N, Karemaker JM, Avolio A. Quantification of wave reflection in the human aorta from pressure alone: a proof of principle. *Hypertension* 2006; 48:595–601.

10 Kööbi T, Kaukinen S, Ahola T, Turjanmaa VM. Non-invasive measurement of cardiac output: whole-body impedance cardiography in simultaneous comparison with thermodilution and direct oxygen Fick methods. *Intensive Care Med* 1997; 23:1132–1137.

11 Koskela JK, Tahvanainen A, Haring A, Tikkakoski AJ, Ilveskoski E, Viitala J, *et al.* Association of resting heart rate with cardiovascular function: a cross-sectional study in 522 Finnish subjects. *BMC Cardiovasc Disord* 2013; 13:102.

12 Kööbi T, Kähönen M, Koskinen M, Kaukinen S, Turjanmaa V. Comparison of bioimpedance and radioisotope methods in the estimation of extracellular water volume before and after coronary artery bypass grafting operation. *Clin Physiol* 2000; 20:283–291.

13 Kööbi T, Kähönen M, Iivainen T, Turjanmaa V. Simultaneous non-invasive assessment of arterial stiffness and haemodynamics–a validation study. *Clin Physiol Funct Imaging* 2003; 23:31–36.

14 Wilenius M, Tikkakoski AJ, Tahvanainen AM, Haring A, Koskela J, Huhtala H, *et al.* Central wave reflection is associated with peripheral arterial resistance in addition to arterial stiffness in subjects without antihypertensive medication. *BMC Cardiovasc Disord* 2016; 16:131.

15 Wu C-H, Yang Y-W, Hung S-C, Tsai Y-C, Hu Y-H, Lin Y-H, *et al.* Effect of treatment on body fluid in patients with unilateral aldosterone producing adenoma: adrenalectomy versus spironolactone. *Sci Rep* 2015; 5:15297.
